# The impact of bleeding on outcomes following lung transplantation: a retrospective analysis using the universal definition of perioperative bleeding

**DOI:** 10.1186/s13019-024-02952-z

**Published:** 2024-07-25

**Authors:** Kevin A. Wu, Joshua K. Kim, Morgan Rosser, Bryan Chow, Brandi A. Bottiger, Jacob A. Klapper

**Affiliations:** 1 Duke School of Medicine, Durham, NC USA; 2 Duke Division of Cardiovascular and Thoracic Surgery, Duke University School of Medicine, 2301 Erwin Rd, 27710 Durham, NC USA; 3 Division of Cardiothoracic Anesthesiology, Duke University, Durham, NC USA

**Keywords:** Lung transplantation, Transfusions, Perioperative bleeding, Primary graft dysfunction, Patient outcomes, Risk factors

## Abstract

**Background:**

Lung transplantation (LT) represents a high-risk procedure for end-stage lung diseases. This study describes the outcomes of patients undergoing LT that require massive transfusions as defined by the universal definition of perioperative bleeding (UDPB).

**Methods:**

Adult patients who underwent bilateral LT at a single academic center were surveyed retrospectively. Patients were grouped by insignificant, mild, or moderate perioperative bleeding (insignificant-to-moderate bleeders) and severe or massive perioperative bleeding (severe-to-massive bleeders) based on the UDPB classification. Outcomes included 1-year survival and primary graft dysfunction (PGD) of grade 3 at 72 h postoperatively. Multivariable models were adjusted for recipient age, sex, body mass index (BMI), Lung allocation score (LAS), preoperative hemoglobin (Hb), preoperative extracorporeal membrane oxygenation (ECMO) status, transplant number, and donor status. An additional multivariable model was created to find preoperative and intraoperative predictors of severe-to-massive bleeding. A *p*-value less than 0.05 was selected for significance.

**Results:**

A total of 528 patients were included, with 357 insignificant-to-moderate bleeders and 171 severe-to-massive bleeders. Postoperatively, severe-to-massive bleeders had higher rates of PGD grade 3 at 72 h, longer hospital stays, higher mortality rates at 30 days and one year, and were less likely to achieve textbook outcomes for LT. They also required postoperative ECMO, reintubation for over 48 h, tracheostomy, reintervention, and dialysis at higher rates. In the multivariate analysis, severe-to-massive bleeding was significantly associated with adverse outcomes after adjusting for recipient and donor factors, with an odds ratio of 7.73 (95% CI: 4.27–14.4, *p* < 0.001) for PGD3 at 72 h, 4.30 (95% CI: 2.30–8.12, *p* < 0.001) for 1-year mortality, and 1.75 (95% CI: 1.52–2.01, *p* < 0.001) for longer hospital stays. Additionally, severe-to-massive bleeders were less likely to achieve textbook outcomes, with an odds ratio of 0.07 (95% CI: 0.02–0.16, *p* < 0.001). Preoperative and intraoperative predictors of severe/massive bleeding were identified, with White patients having lower odds compared to Black patients (OR: 041, 95% CI: 0.22–0.80, *p* = 0.008). Each 1-unit increase in BMI decreased the odds of bleeding (OR: 0.89, 95% CI: 0.83–0.95, *p* < 0.001), while each 1-unit increase in MPAP increased the odds of bleeding (OR: 1.04, 95% CI: 1.02–1.06, *p* < 0.001). First-time transplant recipients had lower risk (OR: 0.16, 95% CI: 0.06–0.36, *p* < 0.001), whereas those with DCD donors had a higher risk of severe-to-massive bleeding (OR: 3.09, 95% CI: 1.63–5.87, *p* = 0.001).

**Conclusion:**

These results suggest that patients at high risk of massive bleeding require higher utilization of hospital resources. Understanding their outcomes is important, as it may inform future decisions to transplant comparable patients.

## Background

Lung transplantation (LT) is a complex procedure for patients with end-stage lung disease that offers hope and a survival benefit [1]. LT survival, however, has lagged compared to other solid organ transplantations [2, 3]. 

The decision to transplant a patient entails consideration of many factors, including the severity of lung disease, efficacy of alternative treatments, the patient’s general health status, and likelihood of success [4]. As demand for LT increases, selecting recipients who will maximally benefit is necessary, [5] and selection criteria should consider patient demographics, comorbidities, and potential complications [6]. 

Perioperative bleeding during LT and the need for transfusion poses significant risks to transplanted organs [7]. For example, a growing body of evidence suggests that large-volume transfusion negatively impacts graft function (i.e. primary graft dysfunction (PGD)) which may translate into chronic allograft dysfunction (CLAD), and shorten overall survival. Consequently, understanding the preoperative risks for bleeding before LT can help inform decisions about whether or not to transplant an individual.

Increased bleeding, as defined by the universal definition of perioperative bleeding (UDPB), requires additional blood products and is associated with increased morbidity and mortality in various cardiac surgical settings [8]. In this study, we applied this definition of massive bleeding retrospectively to our LT cohort in order to describe the impact of increased bleeding on outcomes for those recipients based on the UDPB. In some instances, bleeding is unanticipated but often it can be predicted based on the patient’s history or current clinical situation. It is our hope that the insights from this retrospective analysis will inform clinical decision-making around the appropriateness of LT in patients at high risk of increased bleeding and transfusion.

## Methods

### Patients and study design

This was a retrospective cohort study of patients undergoing bilateral orthotopic lung transplantations (BOLT) at a single-center, large-volume LT program. Approval from the Institutional Review Board (IRB) was obtained prior to the initiation of this study (Pro00093325). Patients were included in the cohort if they were identified as undergoing LT between January 1, 2017, and July 31, 2022. Multi-visceral transplantation patients and patients who underwent a single orthotopic LT were excluded. The final cohort (*n* = 528) contained patients over 18 years old who underwent BOLT. The UDPB was used to separate patients into two groups: patients with insignificant, mild, or moderate bleeding (insignificant-to-moderate bleeders) (*n* = 357) and patients with severe or massive bleeds (severe-to-massive bleeders) (*n* = 171) [9]. The UDPB stratifies patients into five classes based on nine clinical events that occur during surgery or within the first postoperative day, including blood loss, delayed sternal closure, and need for blood products. Patients were dichotomized by groups; classes 0 to 2 were insignificant-to-moderate bleeders, and classes 3 and 4 were categorized as severe-to-massive bleeders. Patients were divided into two groups to emphasize severity and acuity of presentation, enabling clearer comparisons of outcomes. Furthermore, this dichotomization reduced the comparison groups to allow for enhanced statistical analysis.

### Variables

Characteristics for lung recipients were collected, including age, gender, body mass index (BMI), preoperative diagnosis, pulmonary hypertension, mean pulmonary artery pressure (MPAP), lung allocation score (LAS), previous lung surgery, including prior LT, or pleural procedure, left ventricular ejection fraction, preoperative extracorporeal membrane oxygenation (ECMO), preoperative ventilation status, and preoperative labs including the International Normalized Ratio (INR), creatine (Cr), hemoglobin (Hb), and platelet counts. Donor variables such as age, gender, BMI, smoking status, donation after circulatory death (DCD), donation after brain death (DBD), and a medical history of hypertension or cancer were also collected. Additionally, operative characteristics, including the use and type of extracorporeal life support (ECLS) such as venovenous (VV) ECMO and venoarterial (VA) ECMO, duration of ECLS, duration of total ischemia, the intraoperative administration of tranexamic acid (TXA), and the presence of intraoperative complications were recorded.

At our institution, the algorithm for mechanical support during LT primarily involves routine use of VA ECMO, while CPB is reserved for emergencies or cases involving concomitant heart surgery [10]. Anticoagulation is managed using activated clotting time targets of 180–220 s for VA ECMO and > 400 s for CPB, with additional monitoring of platelet counts, fibrinogen levels, and rotational thromboelastometry (ROTEM) at the first lung reperfusion.

### Outcomes

The primary outcome of interest was the occurrence of PGD grade 3 at 72 h after LT. PGD was defined according to the International Society for Heart and Lung Transplantation [8]. Several secondary outcomes were analyzed in relation to UDPB classification, including one-year mortality, hospital length of stay (LOS), and textbook outcome which is a composite outcome measure that aggregates intraoperative complications, need for postoperative interventions, readmission to the ICU or hospital within 30 days, length of stay exceeding the 75th percentile for LT patients, mortality within 90 days, acute rejection within 30 days, grade 3 primary graft dysfunction at 48–72 h, use of postoperative extracorporeal membrane oxygenation, tracheostomy within 7 days, inpatient dialysis, reintubation, and extubation more than 48 h after the transplant [11, 12]. The measurement of textbook outcome has been associated with improved post-transplant survival and reliability in center-level LT performance [13].

UDPB classification of bleeding assesses hemostasis, need for blood products, need for surgical reexploration, and salvage treatment [9]. Clinical bleeding evidence, laboratory evidence of coagulopathy, and established transfusion algorithms guided the decision to transfuse blood products or perform further intervention [14].

### Statistical analyses

Descriptive statistics were calculated to provide a summary of the cohort’s outcomes, with frequencies and percentages used to summarize categorical variables. Continuous variables were compared using the Mann-Whitney U test and independent samples t-test. The Mann-Whitney U test compared medians of two independent groups when data deviated from normality or sample sizes were small, while the independent samples t-test compared means under normality and homogeneity of variance assumptions. The chi-squared test and Fisher exact test examined associations between categorical variables, with the Fisher exact test applied to small sample sizes or violated chi-square test assumptions. Univariate and multivariable regression models for the bleeding groups were performed on outcomes including textbook outcomes, PGD grade 3 at 72 h, one-year mortality, and hospital LOS. Multivariable logistic regression models were adjusted for recipient age, sex, BMI, LAS, group diagnosis, preoperative ECMO status, number of transplants, preoperative Hb, and donor status (DCD vs. DBD). Variables were selected by fellowship trained cardiothoracic surgeons and anesthesiologist based on clinical relevance and pertinence to outcomes of interest.

Additionally, univariate analysis evaluated the significance of these variables. Multicollinearity between variables in the multiple regression models was assessed using the variance inflation factor (VIF). All VIFs were below 3, indicating low multicollinearity, which was not considered an issue. Variables were used in their recorded format whenever possible. If a typically continuous variable was summarized as categorical, it was due to differing recording methods between data sources, ensuring the most complete dataset with the lowest rate of missingness.

Missing data rates were monitored, with the highest rate at 9% (LV Eject) in the Recipient table, while all other rates were below 5%, making imputation unnecessary. Preoperative ECMO data were intentionally missing due to non-use. PGD, textbook outcomes, and mortality outcomes had no missing data, and only 4 cases (< 1%) lacked hospital discharge dates, which excluded them from hospital length of stay regression models.

We performed an additional multivariable logistic regression analysis to find predictors of severe-to-massive bleeding. A stepwise selection was performed to identify the most significant predictors for severe-to-massive bleeding. Initially, a model with no predictors was considered and a full model including all variables from the recipient table (Table 1), as well as Donor Status (DBD vs. DCD) and total ischemic time. Intraoperative variables were excluded to avoid conceptual overlap with UDPB status. Stepwise selection iteratively added and removed variables to determine the best prediction model based on the Akaike Information Criterion (AIC) [15]. The final model included recipient race (White vs. Black), BMI, MPAP, preoperative ECMO, first transplant status, and Donor Status (DCD vs. DBD) as significant predictors. Statistical significance was determined by a *p*-value less than 0.05, with analysis conducted by a certified statistician in R version 4.2.2 [16–20].

**Table 1:** Demographic and clinical characteristics of recipients

Characteristics	Insignificant-to-Moderate Bleeders (*n* = 357)	Severe-to-Massive Bleeders (*n* = 171)	*P*-value
**Transplant Year** (%)			0.893
2017	50 (14.0)	24 (14.0)	
2018	51 (14.3)	20 (11.7)	
2019	73 (20.4)	37 (21.6)	
2020	76 (21.3)	43 (25.1)	
2021	70 (19.6)	31 (18.1)	
2022	37 (10.4)	16 (9.4)	
**Age** (median [IQR])	63.00 [54.00, 68.00]	55.00 [37.50, 64.00]	< 0.001
**Sex**(%)			0.039
Female	142 (39.8)	85 (49.7)	
Male	215 (60.2)	86 (50.3)	
**Race** (%)			0.078
Black or African American	36 (10.2)	26 (15.7)	
Caucasian/White	314 (88.7)	140 (84.3)	
Other	4 (1.1)	0 (0.0)	
**BMI** (mean (SD))	24.85 (3.74)	23.26 (3.83)	< 0.001
**Diagnosis** (%)			0.007
A: OBSTRUCTIVE LUNG DISEASE	75 (21.1)	32 (18.7)	
B: PULMONARY VASCULAR DISEASE	5 (1.4)	12 (7.0)	
C: CYSTIC FIBROSIS/IMMUNODEFICIENCY	30 (8.4)	16 (9.4)	
D: RESTRICTIVE LUNG DISEASE	246 (69.1)	111 (64.9)	
**Preop RVF** (%)			0.029
MILD	77 (21.8)	32 (19.4)	
MODERATE	29 (8.2)	17 (10.3)	
NORMAL	243 (68.6)	106 (64.2)	
SEVERE	5 (1.4)	10 (6.1)	
**LV Eject** (%)			0.944
<=50	97 (29.7)	46 (30.5)	
> 55	230 (70.3)	105 (69.5)	
**Pulmonary Hypertension** (%)	165 (46.5)	88 (54.3)	0.119
**MPAP** (median [IQR])	25.00 [20.00, 30.00]	26.00 [22.00, 32.00]	0.01
**CO** (median [IQR])	5.60 [4.90, 6.50]	5.40 [4.60, 6.32]	0.047
**PCWP** (median [IQR])	10.00 [7.00, 13.00]	10.00 [8.00, 13.00]	0.204
**Lung Allocation Score** (mean (SD))	46.72 (12.55)	54.14 (20.21)	< 0.001
**Outpatient** (%)	297 (83.2)	117 (68.4)	< 0.001
**Preoperative Ventilation** (%)	5 (1.4)	28 (16.4)	< 0.001
**Preoperative ECMO** (%)	5 (1.4)	28 (16.4)	< 0.001
**Hemoglobin** (median [IQR])	12.60 [11.47, 13.70]	11.80 [9.85, 13.10]	< 0.001
**Platelets** (median [IQR]), x10ˆ8/L	11.90 [11.30, 12.80]	12.20 [11.30, 13.38]	0.038
**INR** (median [IQR])	1.00 [1.00, 1.10]	1.00 [1.00, 1.10]	0.132
**Creatinine** (median [IQR])	0.90 [0.70, 1.00]	0.90 [0.65, 1.10]	0.757
**Number of Previous Transplants** (%)			< 0.001
0	346 (97.2)	144 (85.7)	
1	8 (2.2)	21 (12.5)	
2	2 (0.6)	3 (1.8)	

Categorical variables are presented as counts and percentages, and continuous variables are presented as median [interquartile range] or mean (standard deviation). Statistical significance was determined using the Chi-Square test for categorical variables, the Mann-Whitney U-Test for non-normally distributed continuous variables, and Independent Samples T-test for normally distributed continuous variables.

Abbreviations: BMI, body mass index; RVF, right ventricular failure; LV, left ventricular; MPAP, mean pulmonary artery pressure; CO, cardiac output; PCWP, pulmonary capillary wedge pressure; ECMO, extracorporeal membrane oxygenation; IQR, interquartile range; SD, standard deviation; INR, international normalized ratio.

## Results

### Patient cohort

**Fig. 1:**
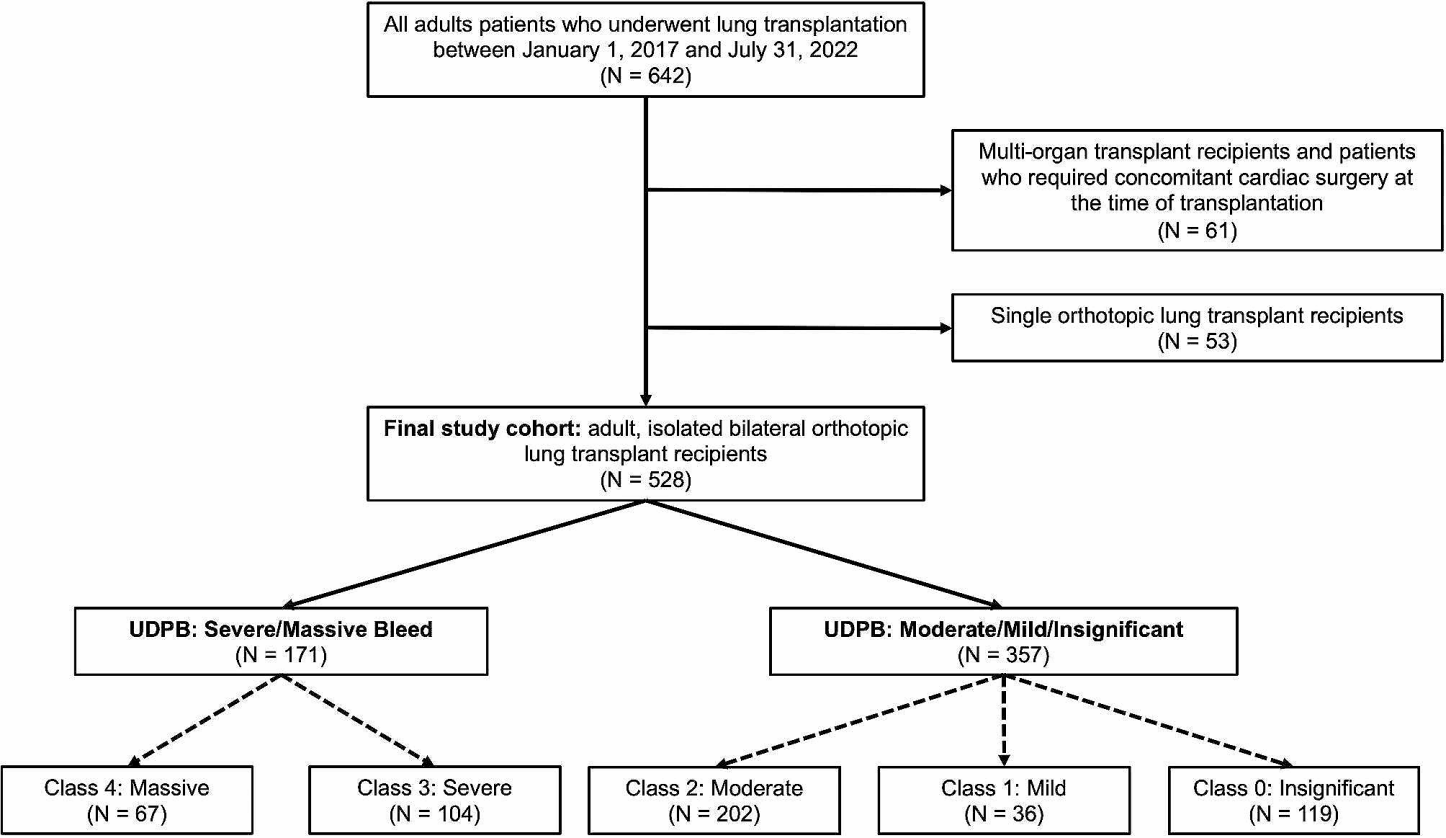
Patient Cohort Selection

There were 642 patients who underwent LT, with 61 patients excluded for undergoing multi-visceral transplantation and 53 excluded for receiving a single orthotopic LT. In total, 528 patients fulfilled inclusion criteria, with 357 insignificant-to-moderate bleeders and 171 severe-to-massive bleeders (Fig. 1). Within the study cohort, there were 432 who had a clamshell incision and 7 patients who underwent a sternotomy. Postoperatively, there were 71 patients who had an open chest requiring delayed closure and 429 patients without delayed closure. Among all transplant recipients, 301 (57.0%) were male, with an average age of 56.6 [50,67] years old and an average BMI of 24.3 ± 3.8 (Table 1).

### Recipient and donor demographics

Severe-to-massive bleeders were younger, (55.00 [37.50, 64.00] vs. 63.00 [54.00, 68.00]; *p* < 0.001), had lower BMIs (23.26 ± 3.83 vs. 24.85 ± 3.74); *p* < 0.001), and were more often female (49.7% v. 39.8%; *p* = 0.039). There were severe-to-massive bleeders distributed across all the different disease subtypes noted with the most common being restrictive lung disease (64.9%). severe-to-massive bleeders did not significantly differ from insignificant-to-moderate bleeders in terms of race (*p* = 0.078).

Donors for the severe-to-massive bleeders had higher rates of DCD compared to the donors for the insignificant-to-moderate bleeders (22.2% vs. 10.7%; *p* = 0.001) (Table 2). Other demographic data, including donor sex (*p* = 0.769), age (*p* = 0.474), BMI (*p* = 0.689), history of hypertension (*p* = 0.394), and history of cancer (*p* = 0.735) were similar between bleeding groups.

**Table 2:** Donor demographic and clinical characteristics

Characteristics	Insignificant-to-Moderate Bleeders (*n* = 357)	Severe-to-Massive Bleeders (*n* = 171)	*P*-value
**Status** (%)			0.001
DBD	318 (89.3)	133 (77.8)	
DCD	38 (10.7)	38 (22.2)	
**Sex** (%)			0.769
Female	138 (39.9)	64 (38.1)	0.769
Male	208 (60.1)	104 (61.9)	
**Age** (median [IQR])	34.00 [26.00, 46.00]	32.50 [27.00, 44.00]	0.474
**BMI** (mean (SD))	27.56 (6.00)	27.33 (6.29)	0.689
**Had Hypertension** (%)	96 (27.9)	40 (24.0)	0.394
**Had Cancer** (%)	6 (1.7)	4 (2.4)	0.735

Tests to determine significance: Chi-Square tests for categorical variables, t-test for continuous. Presence of hypertension and cancer compared using Fisher Exact Test. Median age compared using Mann-Whitney U-Test. Mean BMI compared using Independent Samples T-test.

Abbreviations: N: sample size, IQR: interquartile range, BMI: body mass index, SD: standard deviation, DBD: Donation after Brain Death, DCD: Donation after Circulatory Death.

### Preoperative status

Severe-to-massive bleeders had higher rates of pulmonary vascular disease (7.0% v. 1.4%; *p* = 0.007) defined as mean pulmonary arterial pressures over 25 mmHg, higher mean pulmonary artery pressures (26.00 [22.00, 32.00] mmHg vs. 25.00 [20.00, 30.00] mmHg; *p* = 0.010), and higher rates of moderate (10.3% vs. 8.2%) and severe (6.1% vs. 1.4%) right ventricular dysfunction (*p* = 0.029). Conversely, there was no difference in the left ventricular ejection fraction between the two groups (*p* = 0.933). Severe-to-massive bleeder patients had higher lung allocation scores 54.14 ± 20.21 vs. 46.72 ± 12.55; *p* < 0.001) and were more frequently receiving their second (12.5% vs. 2.2%) or third (1.8% v. 0.6%) transplant (*p* < 0.001). Severe-to-massive bleeders were more likely to require preoperative ventilation (16.4% vs. 1.4%; *p* < 0.001) and less likely to present as an outpatient (68.4% vs. 83.2%; *p* < 0.001). ECMO bridging was more often required for severe-to-massive bleeders (16.4% vs. 1.4%; *p* < 0.001). The severe-to-massive bleeding group was more frequently anemic with lower preoperative Hb (11.80 [9.85, 13.10] vs. 12.60 [11.47, 13.70]; *p* < 0.001) but INR (*p* = 0.132) and creatinine (*p* = 0.757) labs. The severe-to-massive bleeding group had slightly higher median platelet counts compared to the insignificant-to moderate bleeders (12.20 [11.30, 13.38] vs. 11.90 [11.30, 12.80]; *p* = 0.038).

### Intraoperative support

Severe-to-massive bleeders experienced a higher median total ischemic time (484.50 [424.00, 563.25] minutes vs. 432.50 [380.75, 505.25] minutes; *p* < 0.001) (Table 3). They were also more likely to require VV ECMO (7.8% vs. 3.4%) and CPB (14.0% vs. 4.5%) during their operation compared to insignificant-to-moderate bleeders (*p* < 0.001). Intraoperative VA ECMO need was similar between groups. However, severe-to-massive bleeders required intraoperative tranexamic acid (28.1% v. 5.3%; *p* < 0.001) at higher rates and had higher incidences of intraoperative complications (13.5% vs. 1.7%; *p* < 0.001).

**Table 3:** Intraoperative factors

Variable	Insignificant-to-Moderate Bleeders (*n* = 357)	Severe-to-Massive Bleeders (*n* = 171)	*P*-value
**Total Ischemic Time** (mins) (median [IQR])	432.50 [380.75, 505.25]	484.50 [424.00, 563.25]	< 0.001
**Planned Support** (%)			< 0.001
Off Pump	164 (46.3)	53 (32.3)	
On CPB	16 (4.5)	23 (14.0)	
VA ECMO	168 (47.5)	77 (47.0)	
VV ECMO	6 (1.7)	11 (6.7)	
**Max Support** (%)			< 0.001
Off Pump	145 (40.8)	27 (16.3)	
On CPB	17 (4.8)	42 (25.3)	
VA ECMO	180 (50.7)	84 (50.6)	
VAV ECMO	1 (0.3)	0 (0.0)	
VV ECMO	12 (3.4)	13 (7.8)	
**Had Intraoperative Complication** (%)	6 (1.7)	23 (13.5)	< 0.001
**Had TXA** (%)	19 (5.3)	48 (28.1)	< 0.001

Tests to determine significance: Chi-Square tests for categorical variables, Mann-Whitney U-Test for continuous variables. Total Ischemic Time compared using Mann-Whitney U-Test. Planned and Maximum Support classified as Off Pump, On CPB, VA ECMO, VV ECMO, and VAV ECMO, compared using Chi-Square Test. Presence of intraoperative complication and use of TXA compared using Chi-Square Test.

Abbreviations: N: sample size, IQR: interquartile range, CPB: cardiopulmonary bypass, VA: veno-arterial, ECMO: extracorporeal membrane oxygenation, VV: veno-venous, TXA: tranexamic acid.

### Postoperative outcomes

PGD grade 3 at 72 h was more common in severe-to-massive bleeders (32.7% v. 5.9%; *p* < 0.001) (Table 4). Severe-to-massive bleeders had longer hospital stays (40.00 [25.00, 78.00] vs. 20.00 [16.00, 30.00]; *p* < 0.001) and elevated mortality rates at 30 days (7.0% vs. 0.6%; *p* < 0.001) and one year (22.2% vs. 7.3%; *p* < 0.001). This cohort was less likely to achieve textbook outcomes for LT (2.9% vs. 35.6%; *p* < 0.001). Indeed, they were more dependent on postoperative ECMO within 72 h (48.0% vs. 9.2%; *p* < 0.001), reintubation for over 48 h (24.6% vs. 13.4%; *p* < 0.001), or tracheostomy placement (30.4% v. 9.5%; *p* < 0.001). Severe-to-massive bleeders had higher rates of patients among the top quartile for hospital LOS (46.2% vs. 14.0%; *p* < 0.001). This subgroup also required reintervention (62.6% vs. 35.6%; *p* < 0.001) and dialysis (26.9% vs. 4.5%; *p* < 0.001) more frequently. Correspondingly, 90-day mortality were greater (12.9% vs. 1.1%; *p* < 0.001). However, biopsy proven rejection at 30 days (*p* = 0.097) was similar between groups.

**Table 4:** Outcomes by Bleeding Group in lung transplant recipients

Outcomes	Insignificant-to-Moderate Bleeders (*n* = 357)	Severe-to-Massive Bleeders (*n* = 171)	*P*-value
**Primary Graft Dysfunction Grade 3 at 72 h** (%)	21 (5.9)	56 (32.7)	< 0.001
**Hospital Length of Stay** (Days) (median [IQR])	20.00 [16.00, 30.00]	40.00 [25.00, 78.00]	< 0.001
**ICU Length of Stay** (Days) (median [IQR])	4.00 [3.00, 6.00]	3.00 [1.00, 6.00]	< 0.001
**Mortality** (1 Year) (%)	26 (7.3)	38 (22.2)	< 0.001
**Mortality** (30 Days) (%)	2 (0.6)	12 (7.0)	< 0.001
**Textbook Outcome** (%)	127 (35.6)	5 (2.9)	< 0.001
**Reintervention** (%)	31 (8.7)	107 (62.6)	< 0.001
**Biopsy Proven Rejection** (30 Days) (%)	33 (9.2)	8 (4.7)	0.097
**Dialysis/Renal Treatment** (%)	16 (4.5)	46 (26.9)	< 0.001
**Extubated > 48 h Postop** (%)	123 (34.5)	121 (70.8)	< 0.001
**Hospital Readmission** (30 Days) (%)	83 (23.2)	35 (20.5)	0.544
**Reintubation** (%)	48 (13.4)	42 (24.6)	0.002
**ECMO first 72 h Postop** (%)	33 (9.2)	82 (48.0)	< 0.001
**Mortality** (90 Days) (%)	4 (1.1)	22 (12.9)	< 0.001
**Tracheostomy** (7 Days) (%)	34 (9.5)	52 (30.4)	< 0.001
**LOS > 75th Percentile** (%)	50 (14.0)	79 (46.2)	< 0.001

Tests to determine significance: Chi-Square tests for categorical variables, t-test for continuous variables. Outcomes compared between bleeding groups: Insignificant-to-Moderate Bleeders and Severe-to-Massive Bleeders.

Abbreviations: IQR: interquartile range, ICU: intensive care unit, LOS: length of stay, ECMO: extracorporeal membrane oxygenation.

### Regression analysis

**Fig. 2:**
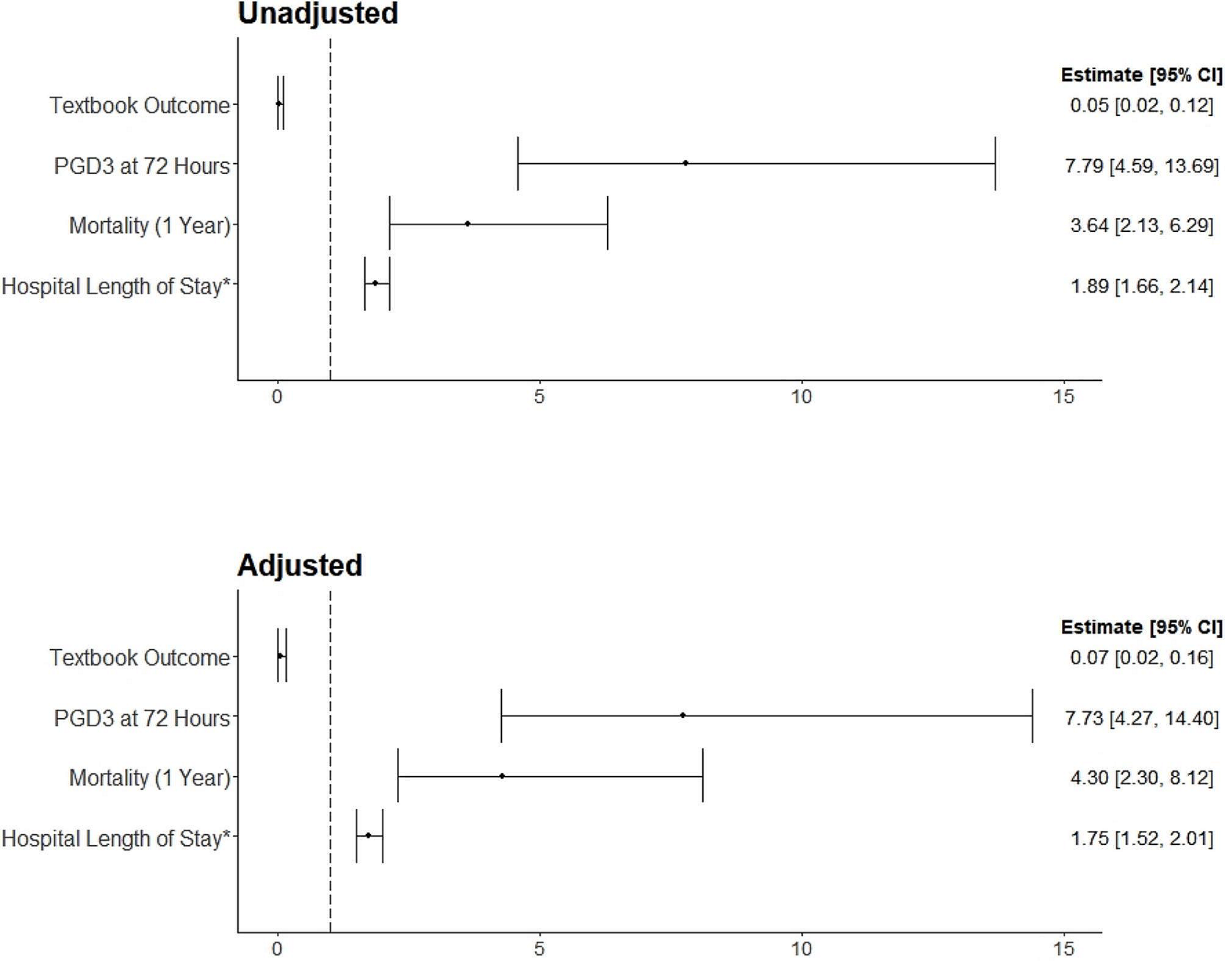
Univariable and Multivariable Analysis for the Odds Ratio of severe-to-massive Bleeders. The multivariable models adjusted for recipient’s age, sex, body mass index, group diagnosis, lung allocation score, preoperative hemoglobin, preoperative ECMO status (Y/N), transplant number (first transplant Y/N), and donor status

Univariate analysis against PGD3 at 72 h showed that relative to insignificant-to-moderate bleeding, severe-to-massive bleeding increased rates of PGD3 at 72 h (OR = 7.79, 95% CI: 4.59–13.69, *p* < 0.001) (Fig. 2). Severe-to-massive bleeding significantly extinguished odds of achieving textbook outcomes, (OR = 0.05, 95% CI = 0.02–0.12, *p* < 0.001), while over tripling risks one-year mortality rate (OR = 3.64, 95% CI = 2.13–6.29, *p* < 0.001) (Table 5). Hospital LOS also showed a significant association, with an odds ratio of 1.89 (95% CI: 1.66–2.14, *p* < 0.001), indicating a longer hospital stays for patients with severe-to-massive bleeding.

**Table 5:** Regression analysis by Bleeding Group in lung transplant recipients

	Univariate				Multivariable				
Outcome	Estimate	95% Confidence		*P*-value	Estimate	95% Confidence		*P*-value	
		Lower	Upper			Lower	Upper		
PGD3 at 72 h	7.79	4.59	13.69	< 0.001	7.73	4.27	14.4	< 0.001	
Textbook Outcome	0.05	0.02	0.12	< 0.001	0.07	0.02	0.16	< 0.001	
Mortality (1 Year)	3.64	2.13	6.29	< 0.001	4.30	2.30	8.12	< 0.001	
Hospital Length of Stay (Days)*	1.89	1.66	2.14	< 0.001	1.75	1.52	2.01	< 0.001	

*Estimate reported is Mean Ratio, all others are odds ratios

Models adjusting for recipient’s age, sex, BMI, Group Diagnosis, LAS, preoperative hemoglobin, preoperative ECMO status (YN), Transplant number (First transplant YN), and Donor Status

Abbreviations: PGD3: Primary Graft Dysfunction Grade 3, BMI: Body Mass Index, LAS: Lung Allocation Score, ECMO: Extracorporeal Membrane Oxygenation

In the multivariate analysis, severe-to-massive bleeding remained significantly associated with adverse outcomes after controlling for confounding characteristics. Severe-to-massive bleeding was independently associated with an increased rate of PGD3 at 72 h (OR = 7.73, 95% CI = 4.27–14.4, *p* < 0.001) and significantly reduced the likelihood of achieving textbook outcomes (OR = 0.07, 95% CI = 0.02–0.16, *p* < 0.001). Being categorized in this bleeding group also raised odds of one-year mortality (OR = 4.30, (95% CI = 2.30–8.12, *p* < 0.001) and was associated with prolonged hospital LOS (OR = 1.75, 95% CI = 1.52–2.01).

There were several preoperative and intraoperative predictors associated with bleeding for the multivariable analysis using bleeding as an outcome. White patients had lower odds of experiencing severe/massive bleeding compared to Black patients (Table 6). Each 1-unit increase in BMI was associated with an 11% reduction in the odds of severe-to-massive bleeding. Additionally, each 1-unit increase in MPAP resulted in a 4% increase in the odds of severe-to-massive. First-time transplant recipients had an 84% lower risk of severe/massive bleeding compared to those undergoing subsequent transplants. Additionally, recipients with DCD donors had a 209% increase in the odds of severe/massive bleeding.

**Table 6:** Multivariable logistic regression analysis with bleeding as an outcome

Variable	OR	95% Confidence		*P*-value
		Lower	Upper	
Recipient Race Caucasian/White vs. AA/Black	0.41	0.22	0.8	0.008
Recipient BMI	0.89	0.83	0.95	< 0.001
MPAP	1.04	1.02	1.06	< 0.001
Preop ECMO Yes vs. No	17.51	5.15	81.62	< 0.001
First Transplant Yes vs. No	0.16	0.06	0.36	< 0.001
Donor: DCD vs. DBD	3.09	1.63	5.87	0.001

AIC for model is 468.37

stepwise selection used to determine predictors for Severe/Massive Bleed vs. Not

Abbreviations: OR: Odds Ratio, AA: African American, BMI: Body Mass Index, MPAP: Mean Pulmonary Artery Pressure, ECMO: Extracorporeal Membrane Oxygenation, DCD: Donation After Circulatory Death, DBD: Donation After Brain Death, AIC: Akaike Information Criterion

## Discussion

Among the 32.4% (*n* = 171/528) of patients in the severe-to-massive group, we discovered higher rates of worse outcomes. Most notably, were more likely to have PGD grade 3, had higher one-year mortality rates, and were less likely to achieve textbook outcomes while still requiring longer hospital stays. This relationship held true for both the univariate and multivariate regression.

Multiple studies have examined the impact of transfusion of different blood products on LT outcomes [21–26]. The current study adds to the current literature by demonstrating the association between increased transfusion requirements and adverse outcomes. The findings of this study highlight the significant impact of massive bleeds on the outcomes of LT recipients. Patients who experienced massive bleeds demonstrated higher rates of PGD, reinforcing the notion that severe bleeding during the perioperative period may be associated with an increased risk of graft dysfunction [27]. Diamond et al., in a multicenter study comprised of 1,255 lung LT recipients across 10 different centers found that the use of over one liter of PRBCs was associated with severe PGD at 48 to 72 h postoperatively [28]. Similarly, requiring over four units of PRBCs has been known to increase rates of grade 3 PGD at 72 h [29]. Preventing and managing massive bleeding in patients who are at higher risk of bleeding is essential as PGD has previously been associated with increase morality extending up to 10 years after LT [30, 31]. The exact mechanisms underlying the development of PGD are not fully understood, but several factors have been implicated in its pathogenesis. One mechanism is ischemia-reperfusion injury [32, 33], which triggers a cascade of inflammatory responses, oxidative stress, and ultimately cell damage and dysfunction [34].

Patients with severe-to-massive bleeds required a higher frequency of reinterventions and dialysis, suggesting severe bleeding may exert systemic complications and impact the overall health of transplant recipients. For example, acute kidney injuries have been associated with LT [35], likely exacerbated by pulmonary insults, ultimately requiring higher rates of dialysis [36–38].

Another important finding was the prolonged LOS observed in patients with massive bleeds, which has been associated with increased mortality up to five years post-transplant [39]. Prolonged stays may be attributed to the complex management of bleeding complications, additional interventions, and the overall compromised clinical condition sustained from massive bleeding episodes. Outside of patient outcomes, prolonged hospitalization drains healthcare resources and contributes to healthcare costs [40, 41].

The optimization of patients prior to transplantation is essential. In lower acuity settings, characteristics such as anemia enjoy higher incidence among severe-to-massive bleeders are worth optimizing. Intraoperatively, bleeding may be managed prophylactically and while it happens. TXA, prothrombin complex concentrate (PCC), factor VIIa, and low-dose heparin infusions represent methods that decrease the risk of bleeding. Some hemostatic interventions may encounter limitations when ECMO is employed, calling for careful risk-benefit analysis. Patients with right ventricular dysfunction and pulmonary hypertension require longer mechanical circulatory support runs, which demands conservative use of medical interventions to prevent coagulation. Balancing bleeding risks and thrombotic complications associated with prolonged mechanical circulatory support is imperative for optimizing outcomes in this challenging patient population. It is important to note that these interventions may have unavoidable interactions that impact outcomes following LT.

Perhaps more important than optimization and intraoperative initiatives to limit bleeding is the careful selection and management of potential LT recipients. The current study may have included patients deemed to have a lower risk of bleeding complications based on preoperative assessments, such as evaluation of anemia, coagulation profiles, and identification of potential bleeding risk factors. These efforts in patient selection and risk stratification may have resulted in a cohort with a lower overall incidence of massive bleeding. To that end, the worse outcome in severe-to-massive bleeders raises pivotal questions: firstly, the appropriateness of the transplantation for these patients, and secondly, the selection between BOLT and single orthotopic lung transplantations (SOLT) procedures. Previous research has demonstrated that although BOLT generally has improved long-term outcomes, short-term outcomes may be better in SOLT, and patients with increased risk of peri-operative mortality (such as high risk of massive bleeds) may benefit from SOLT [42]. The differences in indications between BOLT and SOLT may also contribute to the outcomes demonstrated in the two procedures and future studies should focus on elucidating these interactions. Preoperative assessment and optimization are equally essential and should be considered in conjunction to limit bleeding complications.

A previous study found the rate of massive bleeding ranged between 19% and 33% [22]. Our combined rate of severe and massive bleeding aligns with prior literature, although our incidence of massive bleeding was even lower at 12.9%. The current study’s lower rate of massive transfusion can be explained by several factors. Over time, advancements in surgical techniques and perioperative management of bleeding may have contributed to reduced intraoperative bleeding and subsequent lower transfusion requirements.

This is one of the first studies applying the UDPB to LT [8]. Applying this definition offers several benefits. First, having a standardized definition facilitates consistency and comparability across studies, enhancing the accuracy and reliability of research findings. Downstream, this facilitates the pooling of data from different centers, enabling a more comprehensive analysis of outcomes and the development of evidence-based practices. The UDPB also allows clinicians to classify patients postoperatively and identify patients at higher risk for worse outcomes. By accurately defining and categorizing massive bleeding, the UDPB aids in risk stratification, facilitating the identification of high-risk patients who may require additional monitoring and interventions.

There are several limitations to this current study. This study is retrospective, limiting the conclusions that can be drawn. Second, typically sicker patients (e.g., bridged patients) require higher rates of transfusions, and thus will have worse outcomes postoperatively. Although this could potentially confound the results, the multivariable regression in the study accounted for various factors (e.g., LAS, group diagnosis, etc.) to mitigate the impact of baseline illness severity on outcomes and isolate the impact of bleeding. The study, however, offers the benefit of utilizing our center’s large volume to find a large cohort of patients requiring massive transfusions. Using the UDPB provides the benefit of easily allowing for comparison across different studies and centers.

This study emphasizes the importance of carefully considering the risk of massive bleeding and transfusion in the selection and management of LT recipients. The results underscore the substantial utilization of hospital resources associated with patients at high risk of massive bleeding, including prolonged LOS and increased morbidity and mortality rates. Understanding the outcomes of this high-risk subgroup is critical, as it can inform future decisions regarding the transplantation of comparable patients. Implementing strategies to prevent and effectively manage massive bleeds during LT is vital to optimize patient outcomes and allocate healthcare resources efficiently. Ultimately, this study helps to provide a comprehensive understanding of the associated clinical outcomes, resource utilization, and potential risks, which can inform clinical decision-making and improve patient care in this high-risk population.

## Conclusion

This retrospective cohort study highlights the impact of severe perioperative bleeding, as defined by the UDPB, on outcomes in LT recipients. Severe-to-massive bleeding was associated with higher rates of PGD grade 3 at 72 h, increased 1-year mortality, lower likelihood of achieving textbook outcomes, and longer hospital stays. These findings underscore the importance of careful patient selection and perioperative management to minimize the risk of severe bleeding and its associated complications. Strategies to prevent and manage bleeding in high-risk patients, including optimizing preoperative status and judicious use of transfusions and hemostatic agents, are crucial for improving outcomes in LT. Further research is needed to explore specific interventions and protocols that can reduce the risk of severe bleeding and improve outcomes in this patient population.

## Data Availability

The datasets used and/or analyzed during the current study are available from the corresponding author on reasonable request.

## Abbreviations

UDPB: Universal Definition of Perioperative Bleeding
LT: Lung Transplantation
PGD: Primary Graft Dysfunction
BMI: Body Mass Index
MPAP: Mean Pulmonary Artery Pressure
LAS: Lung Allocation Score
ECMO: Extracorporeal Membrane Oxygenation
INR: International Normalized Ratio
Cr: Creatine
Hb: Hemoglobin
ECLS: Extracorporeal Life Support
VV ECMO: Venovenous Extracorporeal Membrane Oxygenation
VA ECMO: Venoarterial Extracorporeal Membrane Oxygenation
TXA: Tranexamic Acid
LOS: Length of Stay
PRBCs: Packed Red Blood Cells
AKI: Acute Kidney Injury
ROTEM: Rotational Thromboelastometry
